# Supplementary material for: Novel Driver Strength Index highlights important cancer genes in TCGA PanCanAtlas patients
Source: PeerJ. 2022 Aug 11;10:e13860. doi: 10.7717/peerj.13860 (PMC9375969; doi:10.7717/peerj.13860)
Supplement: Supplemental Information 2 [file peerj-10-13860-s002.zip › COHORTS/genes plots/detailed/2021_8_16_14_9_distribution_events_detailed_25.pdf]

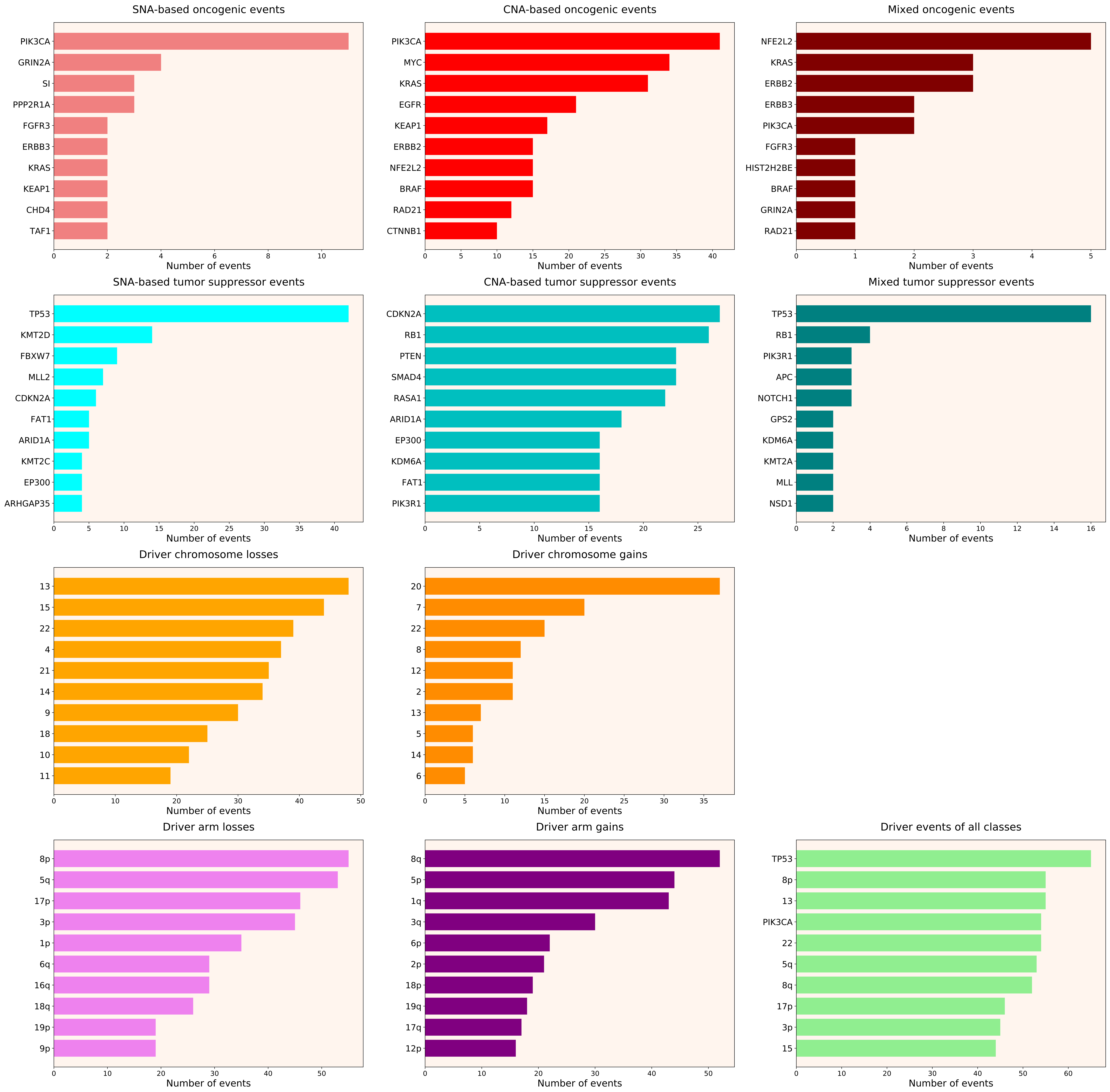

SNA-based oncogenic events
PIK3CA
GRIN2A
SI
PPP2R1A
FGFR3
ERBB3
KRAS
KEAP1
CHD4
TAF1
0 2 4 6 8 10
Number of events
CNA-based oncogenic events
PIK3CA
MYC
KRAS
EGFR
KEAP1
ERBB2
NFE2L2
BRAF
RAD21
CTNNB1
0 5 10 15 20 25 30 35 40
Number of events
Mixed oncogenic events
NFE2L2
KRAS
ERBB2
ERBB3
PIK3CA
FGFR3
HIST2H2BE
BRAF
GRIN2A
RAD21
0 1 2 3 4 5
Number of events
SNA-based tumor suppressor events
TP53
KMT2D
FBXW7
MLL2
CDKN2A
FAT1
ARID1A
KMT2C
EP300
ARHGAP35
0 5 10 15 20 25 30 35 40
Number of events
CNA-based tumor suppressor events
CDKN2A
RB1
PTEN
SMAD4
RASA1
ARID1A
EP300
KDM6A
FAT1
PIK3R1
0 5 10 15 20 25
Number of events
Mixed tumor suppressor events
TP53
RB1
PIK3R1
APC
NOTCH1
GPS2
KDM6A
KMT2A
MLL
NSD1
0 2 4 6 8 10 12 14 16
Number of events
Driver chromosome losses
13
15
22
4
21
14
9
18
10
11
0 10 20 30 40 50
Number of events
Driver chromosome gains
20
7
22
8
12
2
13
5
14
6
0 5 10 15 20 25 30 35
Number of events
Driver arm losses
8p
5q
17p
3p
1p
6q
16q
18q
19p
9p
0 10 20 30 40 50
Number of events
Driver arm gains
8q
5p
1q
3q
6p
2p
18p
19q
17q
12p
0 10 20 30 40 50
Number of events
Driver events of all classes
TP53
8p
13
PIK3CA
22
5q
8q
17p
3p
15
0 10 20 30 40 50 60
Number of events
